# Low-Anxiety Rat Phenotypes Can Be Further Reduced through Genetic Intervention

**DOI:** 10.1371/journal.pone.0083666

**Published:** 2013-12-30

**Authors:** Gabriela Ferreira de Medeiros, Elayne Pereira, Natalli Granzotto, André Ramos

**Affiliations:** Laboratório de Genética do Comportamento, Departamento de Biologia Celular, Embriologia e Genética, Centro de Ciências Biológicas, Universidade Federal de Santa Catarina, Florianópolis, Santa Catarina, Brazil; Radboud University, The Netherlands

## Abstract

**Background:**

A previous study using an intercross between the inbred rat strains Lewis (LEW) and Spontaneously Hypertensive Rats (SHR) identified a *locus* on chromosome 4, named *Anxrr16*, influencing an experimental index of anxiety and showing a transgressive effect, with alleles from the LEW strain (more anxious) decreasing rather than increasing anxiety.

**Objective:**

To confirm the location and isolate the effect of a rat genome region named *Anxrr16* through a planned genomic recombination strategy, where the target *locus* in SHR rats was replaced with LEW genetic material.

**Methods:**

A new congenic strain, named SHR.LEW-*Anxrr16* (SLA16), was developed from a cross between LEW (donor) and SHR (receptor) rats and then evaluated in several anxiety-related tests. The activity and attention levels of the new strain were also evaluated, since hyperactivity was observed during its construction and because SHR is a model of attention deficit hyperactivity disorder.

**Results:**

Significant effects of *Anxrr16* were found for open field central locomotion, as well as for other indices of anxiety from the light/dark box, triple test and T-maze. In all cases, the low-anxiety levels of SHR rats were further reduced by the insertion of LEW alleles. Differences in locomotor activity were found only in unfamiliar (hence stressful) environments and no genetic effects were observed in indices of attention.

**Conclusion:**

The SLA16 strain can help in the identification of the molecular pathways involved in experimental anxiety and it demonstrates how apparently extreme phenotypes sometimes hide major opposite-acting genes.

## Introduction

Anxiety can be defined as a state of fear developed in anticipation to a threat, which promotes vigilance and facilitates avoidance behavior [1]. A distinction has been proposed between trait and state anxiety, with the former being a constant emotional state that can become pathological and the latter being the healthy, momentary consequence of an anxiogenic stimulus [2]. Some authors, however, suggest that trait and state anxiety constitute two sides of the same coin, with trait anxiety being conditioned by the intensity and frequency of anxious-state episodes [3], [4]. When these feelings get excessive they may disrupt life functioning, thus being classified as anxiety disorders [1], the most prevalent lifetime group of disorders in the American population [5].

Given their social impact as well as the genetic factors involved [6], investigating the genetic bases of anxiety is of central importance. Yet, finding genes for anxiety-related traits is difficult, because anxiety is influenced by a myriad of genes (i.e. is polygenic), each of them accounting for only a small proportion of the genetic effects besides being dependent on the environment and on interactions with other genes [7]. Most genetic studies of anxiety, both in humans and animal models, search for anxiety predisposing genes in highly anxious individuals, disregarding the fact that, for being highly polygenic, anxiety-increasing alleles are expected to be found also in non-anxious individuals, just as highly anxious individuals shall carry at least some gene variants that decrease anxiety. In keeping with this conceptual framework, one can assume that the anxiety level of any non-anxious individual is probably not at its lowest possible level. Therefore, in theory, it can still be further reduced through genetic intervention.

In quantitative genetics, transgressive segregation consists in the emergence of phenotypes in the offspring that exceed the values found in the parental generations [8], [9]. A genetic tool that not only provides an example but also can enlighten this phenomenon is the pair of rat strains Lewis (LEW) and Spontaneously Hypertensive Rats (SHR). These inbred strains (where all individuals are genetically identical to each other) contrast in a series of anxiety-related parameters, with LEW displaying a more anxious-like profile than SHR [10], [11]. Using these strains, Ramos et al. identified the first anxiety-related QTL (Quantitative Trait *Locus*) for rats [12]. This genome region, located on chromosome 4 and referred to as *Anxrr16*, was found to affect inner locomotion in the open field, an experimental index of anxiety [13], being the second strongest QTL ever reported for anxiety. Since that seminal study, *Anxrr16* has been repeatedly found to affect the offspring of LEW and SHR strains in a counterintuitive manner, that is, with LEW alleles conferring a lower rather than a higher anxiety profile [14], [15], [16]. In fact, LEW alleles in *Anxrr16* conferred the offspring an even lower anxiety profile than that displayed by the parental strain SHR, showing that this major anxiety QTL had a transgressive effect.

In an attempt to better understand the molecular mechanisms involved in anxiolysis (i.e. the reduction of anxiety), this study aimed to confirm the location and isolate the effect of the rat genome region named *Anxrr16*. This was achieved through the construction of a congenic strain, named SHR.LEW-*Anxrr16* (here abbreviated as SLA16), on which a piece of chromosome 4 containing the *locus* of interest from the LEW strain was inserted into the SHR genome. We hypothesized that the anxiety levels of these congenic animals would be reduced to an even lower level than those from the parental strain SHR, due to the counterintuitive and transgressive nature of *Anxrr16* [12], [14]. Because SHR is also a model of attention deficit hyperactivity disorder (ADHD) [17], [18], possible genetic side effects on motor activity and attention were evaluated.

## Materials and Methods

### Animals

A total of 1274 animals (LEW, SHR and their hybrids) were used for the genetic construction (see below) and 375 rats (SHR and SLA16) were used for phenotyping. SLA16 was fully developed in our laboratory, whereas SHR and LEW had been kept in our laboratory for more than 20 generations under brother-sister mating. All animals were weaned and separated by sex at ∼28 days of age and thereafter kept in collective plastic cages (5–6 rats/cage). A 12-h light/dark schedule (lights on at 7∶00 a.m.) was maintained under a temperature of 22±2°C with food and water available *ad libitum*.

### Ethics Statement

All procedures were conducted according to the guidelines of the local Committee for Animal Care in Research (Comissão de Ética no Uso de Animais - CEUA/UFSC). The protocol was approved by the Committee on the Ethics of Animal Experiments of the Federal University of Santa Catarina/UFSC (Protocol number PP0029 and PP00656). All efforts were made to minimize suffering.

### Genetic Construction and Test Assignment

**Figure 1:**
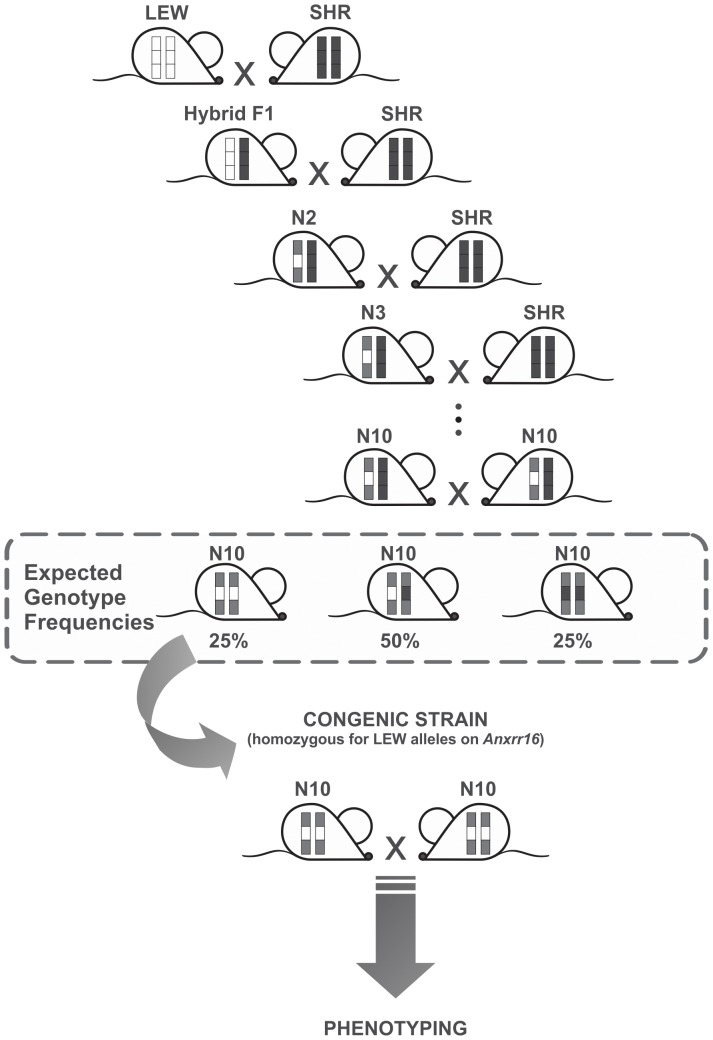
Construction of the SLA16 congenic strain. After obtaining an F1 (or N1), the animals generated were continuously backcrossed to SHR rats for 10 generations and genotyped for three markers (two flanking and one in the middle of *Anxrr16*) for selecting heterozygous animals for the *Anxrr16* region, after which *Anxrr16* heterozygous rats were intercrossed. The congenic strain is homozygous for LEW alleles in the *Anxrr16* region, and about 99.9% SHR in the rest of the genome.

Initially, SHR males (receptors) were intercrossed with LEW females (donors), producing a heterozygous N1 (or F1) generation. N1 was then backcrossed with SHR, generating N2, which was genotyped for a set of molecular markers in the *Anxrr16* region, on chromosome 4. Animals that were heterozygous for these markers were selected for a new backcross with SHR. This process was continuously repeated for ten generations, when N10 animals that were heterozygous for *Anxrr16* were intercrossed to produce an offspring (named N10F1) in which ¼ of the pups were homozygous LEW for the region of interest. Their remaining genome was nearly 99.9% SHR. Congenic SLA16 animals were maintained by intercross and, at each generation (N10F2, N10F3, etc), progenitors were chosen through marker-assisted selection, aiming to increase the frequency of LEW alleles in the *Anxrr16* region and of SHR alleles in the rest of the genome. This genetic construction is represented in Figure 1. In generation N10F4, males and females were submitted between 8 and 12 weeks of age to a battery of tests comprising the open field, light/dark box and unfamiliar activity cages (n = 18–28/sex/strain). At N10F5, male rats 8–10 weeks old were first tested in the open field under either high or low illumination (n = 15–16/strain/light condition) and then divided into three groups to be tested in the T-maze (11–13 weeks, n = 10/strain), attentional set-shifting (12–14 weeks, n = 11/strain) or a familiar activity cage (13–15 weeks, n = 10/strain). At N10F6, males were submitted to the triple test (11 weeks, n = 15/strain). In order to complete sample sizes from the previous generation, some N10F6 males were tested in the T-maze (n = 3/strain) and in the attentional set-shifting (n = 6/strain). Females from this generation were tested in the open field at 16–17 weeks of age following the estrous cycle protocol (n = 30/strain), described below. Finally, in N10F7, animals from both sexes (n = 25–30/sex/strain) were tested at 8–9 weeks of age using the home-cage protocol (see below).

### Genotyping

**Figure 2:**
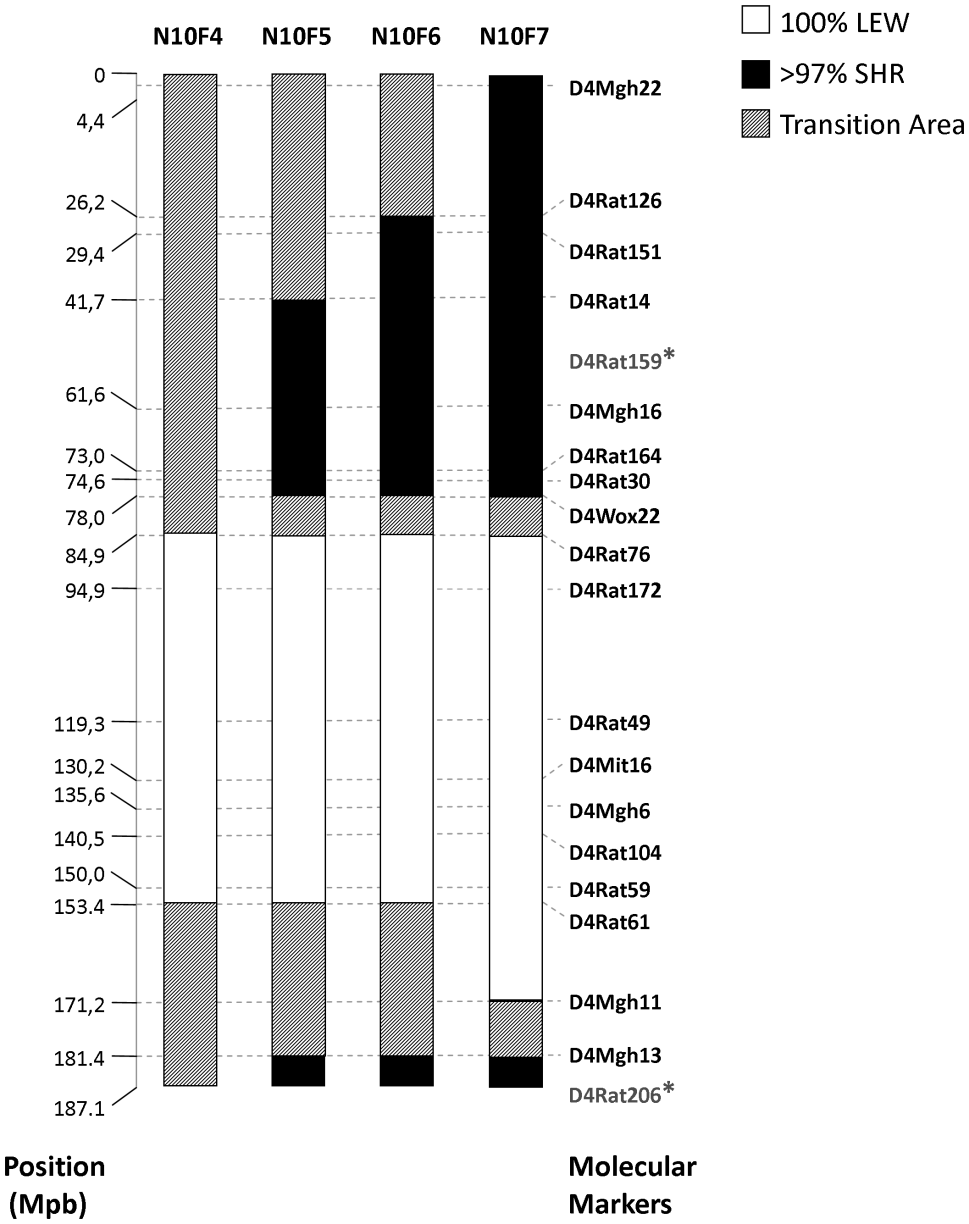
Schematic representation of allele frequencies in SLA16 animals from generations N10F4 to N10F7. The black areas represent portions of the genome where over 97% of the alleles came from the SHR strain, while the white areas are completely constituted by LEW alleles, and the hatched areas (transition) indicate regions where both alleles are found in variable proportions. (*) indicates two molecular markers for which positions in Mpb are not available on the Rat Genome Database, with their locations being estimated based on their position in Centimorgan.

N10F4, N10F5, N10F6 and N10F7 rats were genotyped for 20 polymorphic microsatellite markers mapped on chromosome 4. DNA was extracted from tail tips (sectioned when pups were 1-month old) using *DirectPCR Lysis Reagent (tail)* (Viagen Biotech) and then submitted to polymerase chain reaction (PCR) in a 20-µL reaction volume containing 1 µL of extraction product, 9.5 µL of water, 5 µL of oligonucleotide mix (1 pmol/µL each), 0.38 u of GoTaq® DNA Polymerase, 0.4 µL of dNTP mix (10 mM each) and 4 µL of 5x Green GoTaq® Reaction Buffer (Promega). PCR program was: (i) one cycle at 96°C for 5 min; (ii) 35 cycles at 92°C for 30 s; 51–59°C (depending on the marker) for 1 min, 72°C for 30 s; and (iii) one cycle at 72°C for 2 min. Samples were genotyped in 3% agarose gels stained with ethidium bromide. A representation of SLA16 chromosome 4 is illustrated in Figure 2.

### Phenotyping

A series of behavioral tests were performed to evaluate different aspects of anxiety (open field, light/dark box, T-maze, triple test, home-cage behavior), locomotion (open field, triple test, activity cage, home-cage behavior) and attention-related (attentional set-shifting) behaviors.

#### Open field

The open field apparatus, described by Ramos et al. [11] as a model of anxiety and locomotor activity, consisted of a white square arena (100×100 cm) surrounded by walls (40 cm high), with its floor divided by black lines into 25 squares (20×20 cm) and under a dim illumination of 7 lux. Squares adjacent to the walls formed the “outer” area while the “inner” area consisted of all other squares. The number of squares crossed in each area was registered for 5 min after the animal being positioned in the center of the apparatus. A variant of this protocol compared dim (10 lux) versus bright (530 lux) light conditions (N10F5). Between rats, the apparatus was cleaned with a 10% ethanol solution and paper towels, being this same procedure adopted for all the other tests.

#### Light/Dark box

The light/dark box test, an anxiety model, was conducted as described by Ramos et al. [11]. Briefly, the apparatus consisted on a rectangular box, with its interior divided by a holed wall into two different-sized compartments: one larger (27×27×27 cm), white and strongly illuminated, with its floor divided by black lines into 9 squares (9×9 cm), and one smaller (27×18×27 cm high) and black, with its floor divided by white lines into 6 squares (9×9 cm). Illumination of the light and dark compartments was provided by fluorescent white (9 W) and red (14 W) lamps, located 30 cm above the apparatus floor, providing 1500 and 65 lux, respectively. The rat was placed inside the light compartment and the following measures were registered during 5 min: time spent and number of squares crossed in the light compartment and number of transitions between compartments.

#### T-Maze

The T-maze is thought to access behaviors related to generalized anxiety (through inhibitory avoidance) and panic (through one-way escape) disorders [19]. As described by Ramos et al. [11], the T-maze consisted of four black wooden arms (52 cm elevated from the floor, 50 cm long and 10 cm wide), arranged in a cross-like disposition, with two opposite arms being enclosed (by 40 cm high walls) and two being open (surrounded by a raised ledge, 1 mm thick and 5 mm high). Their intersection formed a central platform (10×13.5 cm) which gave access to any of the arms. One of the enclosed arms was blocked by a black plastic barrier, giving to this plus-shaped maze a T shape, formed by two open arms and one perpendicular enclosed arm (the stem of the T). The protocol was identical to that described by Soares et al. [20]. It included daily handling and habituation to a holding compartment during 5 min for 2 days before the test. In the day before the test, animals were individually confined into one of the open arms for 30 min [21], [22]. On the testing day, each animal was placed in the distal end of the enclosed arm and the time taken to leave this arm with the four paws (baseline avoidance) was registered. This procedure was repeated twice (avoidance 1 and 2), in order to obtain the three scores of inhibitory avoidance. Following that, the animal was placed in the distal end of an open arm, and the time taken to leave it with the four paws (escape 1) was registered. Similarly, two more trials (escape 2 and 3) were carried out for the one-way escape score. Animals were kept in the holding compartment for 30 s between trials and a cutoff time of 300 s was adopted for all trials.

#### Triple test

The triple test, which has been validated as a model of anxiety and locomotor behavior [23], was devised to simultaneously measure a wide range of unconditioned behaviors in only one trial. The apparatus was an integration of three classical anxiety tests: the open field, the elevated plus maze and the light/dark box, all inter-connected in that order, as described by Ramos et al. [23]. The animal was first placed in the center of the open field and allowed to freely explore the apparatus for 15 min, during which classical parameters of anxiety and locomotion from the three individual tests were evaluated, namely the time spent and number of entries made in all protected and unprotected areas.

#### Activity cages

The activity cage consisted of a wooden box (70×27×22 cm high), with a wire-mesh floor and an acrylic lid, equipped with three infrared sensors distributed along its width, which permitted estimating the rat’s locomotor activity through the number of beam interruptions. Animals were placed in the apparatus for either 1 h (unfamiliar condition) or 23.5 h (familiar condition) and their activity scores were registered every 10 min. Under the familiar protocol, food and water were provided *ad libitum* and scores were measured during the final 60 min.

#### Attentional set-shifting

This model of attention was adapted from Floresco et al. [24]. The apparatus consisted of four white wooden arms (60×10 cm), 70 cm elevated from the ground, surrounded by 20 cm walls, and inter-connected by a central platform (10×10 cm), which gave access to any of the arms. Each arm was labeled accordingly to its position (north, east, south or west) in the testing room. Small slots were present at every arm entry, in which a white wooden barrier could be fitted, blocking that entrance and giving the apparatus a T shape. A piece of black-white stripped formica that perfectly fitted an individual arm floor (60×10) was used as a visual cue. One week prior to the beginning of this test, all animals were individually housed, had their food restricted to ∼10 g/day and started to be handled daily for 5 min. On the day before the test, rats were given 10 small food reward pellets in their home-cages, to introduce them to the taste of the reward. **Familiarization:** On the first familiarization day, three food pellets were distributed along each maze’s arm. Animals were placed in the central platform and allowed to freely explore the apparatus and eat all pellets for up to 15 min. A familiarization criterion was set when all the pellets were eaten in less than 10 min and, once achieved, the animal could move on to the next testing phase. Animals who didn’t achieve this criterion were submitted to one more familiarization trial in the following day. **Turn bias determination:** In the day after the criterion was achieved, one of the maze’s arms was blocked and one reward pellet was placed in the distal part of both left and right arms, with the visual cue pseudorandomly placed in one of them. In one trial, animals were placed in the distal part of the stem arm and allowed to turn either left or right to consume the food. After the pellet was consumed, the rat was again placed in the stem arm and allowed to make the next choice. The trial ended after the rat had chosen both arms and eaten the two pellets. After seven trials, the first direction chosen in four or more trials was considered this animal’s turn bias. **Response discrimination task:** The animals were placed in the stem arm and required to turn to the opposite direction of their turn bias, regardless of the visual cue location, to obtain the food reward. The rat’s starting arm was pseudorandomly alternated between west, south and east, in order to discourage it from using visual cues existent in the testing room. The learning criterion in this stage was to turn ten consecutive times to the right side, plus and an eleventh time (probe trial) with north as the starting arm and the visual cue placed in the rat’s turn bias. Animals who got to the probe trial and missed it, were required to make five more consecutive correct choices, followed by another probe trial, until the task was learned. **Visual cue discrimination:** On the following day, a similar procedure was performed, but now with the reward pellet always placed on the arm containing the visual cue, which was located pseudorandomly in left or right arms. In order to obtain the reward, the animal had to change the previous acquired strategy. The same learning criterion from response discrimination task was adopted and, for both discrimination tasks, a maximum cutoff of 100 trials was set. Entries in the arms that did not contain the visual cue were considered errors. Three subtypes of errors were identified as described by Floresco et al. [24]. Perseverative errors reflected the animal’s ability to shift from the first learned strategy, while regressive and never-reinforced errors were an indicative of its capacity of maintaining the new strategy.

#### Home-Cage behavior

N10F7 and SHR rats, separated by sex and strain, were taken with their home-cages (5 rats/cage; 5–6 cages/strain/sex) into the testing room at 10∶00 a.m., and their behavior was registered through a video camera positioned above the cages, without the presence of the experimenter. Recordings happened twice a day in two different 6-hour periods, from 4∶00 to 10∶00 p.m. and from 4∶00 to 10∶00 a.m. Each of these periods had 3 h of darkness (7–10 p.m. or 4–7 a.m.) and 3 h of light (4–7 p.m. and 7–10 a.m.). For the two “darkness blocks”, a red lamp (40 W) was installed above the cages to allow visual observation. Four home-cages (2 cages/strain), positioned side by side, were observed in each recording day. The scan methodology was used for behavioral registration, which consisted of instantaneous observations every 5 min. The following behaviors were registered: drinking, eating, locomotion (or ambulation), grooming, scratching, resting and social interaction. At each scan, the number of rats in the cage that were performing a certain behavior was registered. The sum of these numbers for each 3 h block was taken as the frequency of that behavior and then transformed into percentage, as described by Hinojosa et al. [25].

#### Estrous cycle protocol

In order to test the effects of estrous on the genetic differences between strains [16], female rats were injected subcutaneously with estradiol benzoate (0.1 mg/ml, 0.1 ml per rat) and, 48 h later, with progesterone (5 mg/ml, 1 ml per kg). Both drugs were diluted in sunflower oil. Animals from the estrous-metestrous (EM) group were tested 24 h after receiving progesterone, while those from the diestrous-proestrous (DP) group were tested 96 h after the last injection. Cycle phase was confirmed by microscopic observation of vaginal smears collected after the open field test, dropped over a slide, stained with methylene blue and observed under an optic microscope (10x and 40x).

### Statistics

All analyses were carried out using Statistica 10 software package (Statsoft, Tulsa, OK, USA). Experiments involving only strain as the grouping variable were analyzed through a T-test, whereas a factorial ANOVA was used when two grouping variables were present. The longitudinal T-maze and home-cage behavior data were analyzed by repeated-measures ANOVA. P values <0.05 were considered significant.

## Results

### Construction of the Congenic Strain Revealed only Mild Behavioral Changes

**Figure 3:**
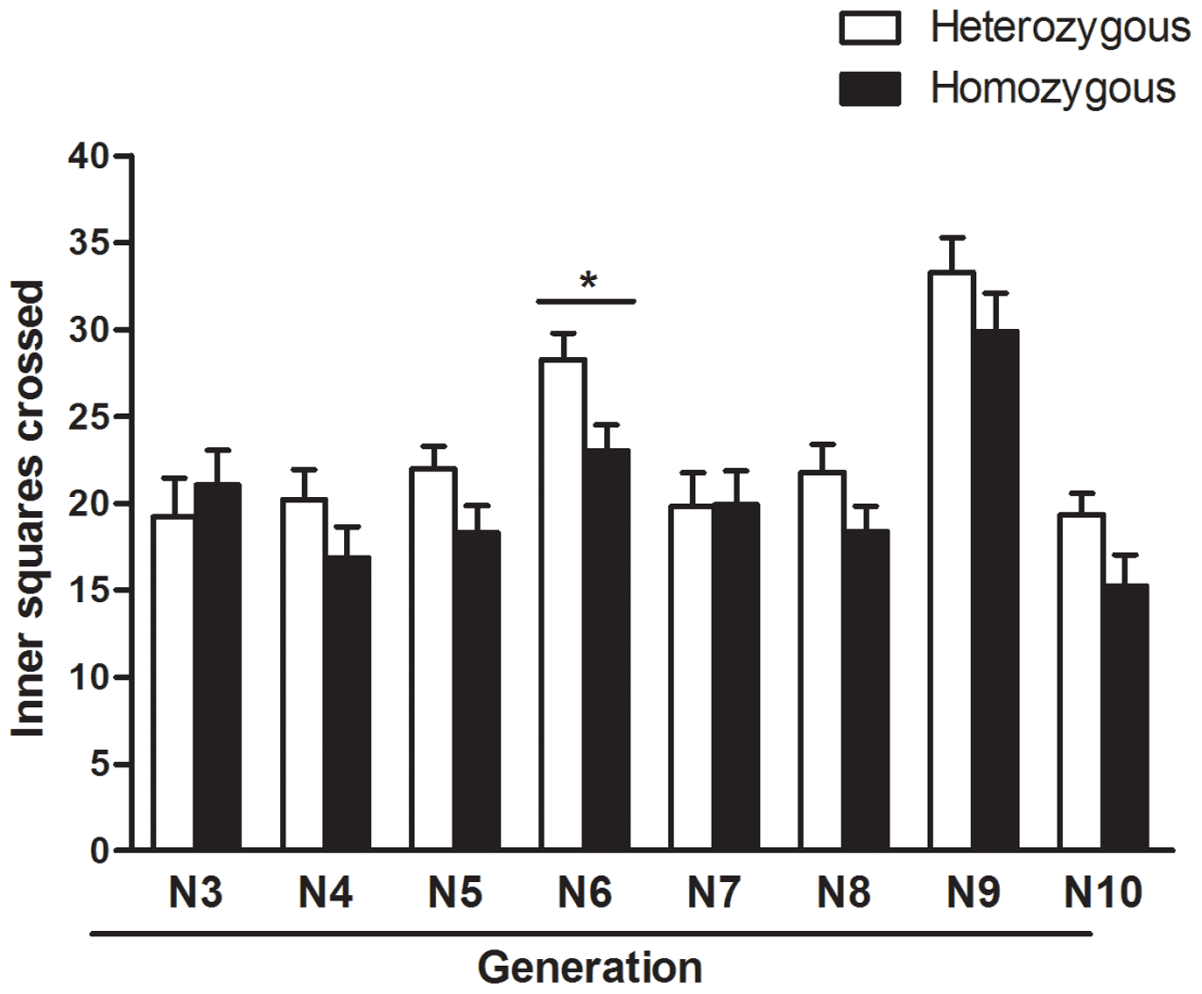
Behavioral profile of the SLA16 congenic strain during its construction. Inner locomotion displayed by animals during the development of SLA16 (generations N3 to N10) in the open field test. In each of these generations, males and females that were either homozygous for SHR alleles or heterozygous in the *Anxrr16 locus* were tested. Bars and vertical lines represent the means and S.E.M. of animals grouped by genotype. (*) represents significant (p<0.05) overall effect of genotype (two-way ANOVA for each generation separately).

In the open field (Figure 3) the two-way ANOVA (for each generation separately) revealed that heterozygous rats of the N6 generation displayed higher locomotion in the inner and aversive area than homozygous SHR rats (F_(1,63)_ = 6.37, p<0.05). Non-significant trends in the same direction were observed in N4, N5 and N8–10, suggesting slightly reduced anxiety levels during development of the new strain. Overall sex differences (not shown) were found for this measure, with females showing higher scores in all generations (p<0.05).

### Genomic Recombination Diminished Anxiety- and Increased Locomotion-related Behaviors of SHR Rats

Once the new strain was 100% homozygous (LEW-LEW) for the *locus* of interest, a two-way ANOVA revealed that SLA16 rats displayed higher locomotion in the inner and aversive area of the open field than SHRs (F_(1,90)_ = 34.07, p<0.001), regardless of sex, suggesting a reduced anxiety in the former [4]. A difference in the same direction (less visible in males) was found for outer locomotion (F_(1,90)_ = 14.11, p<0.001), a measure of motor activity. Overall sex differences were found for both measures, with females presenting higher scores. The open field test performed under either dim or bright light showed no effects of illumination on the evaluated behaviors, though strain differences were maintained regardless of light intensity (data not shown).

**Figure 4:**
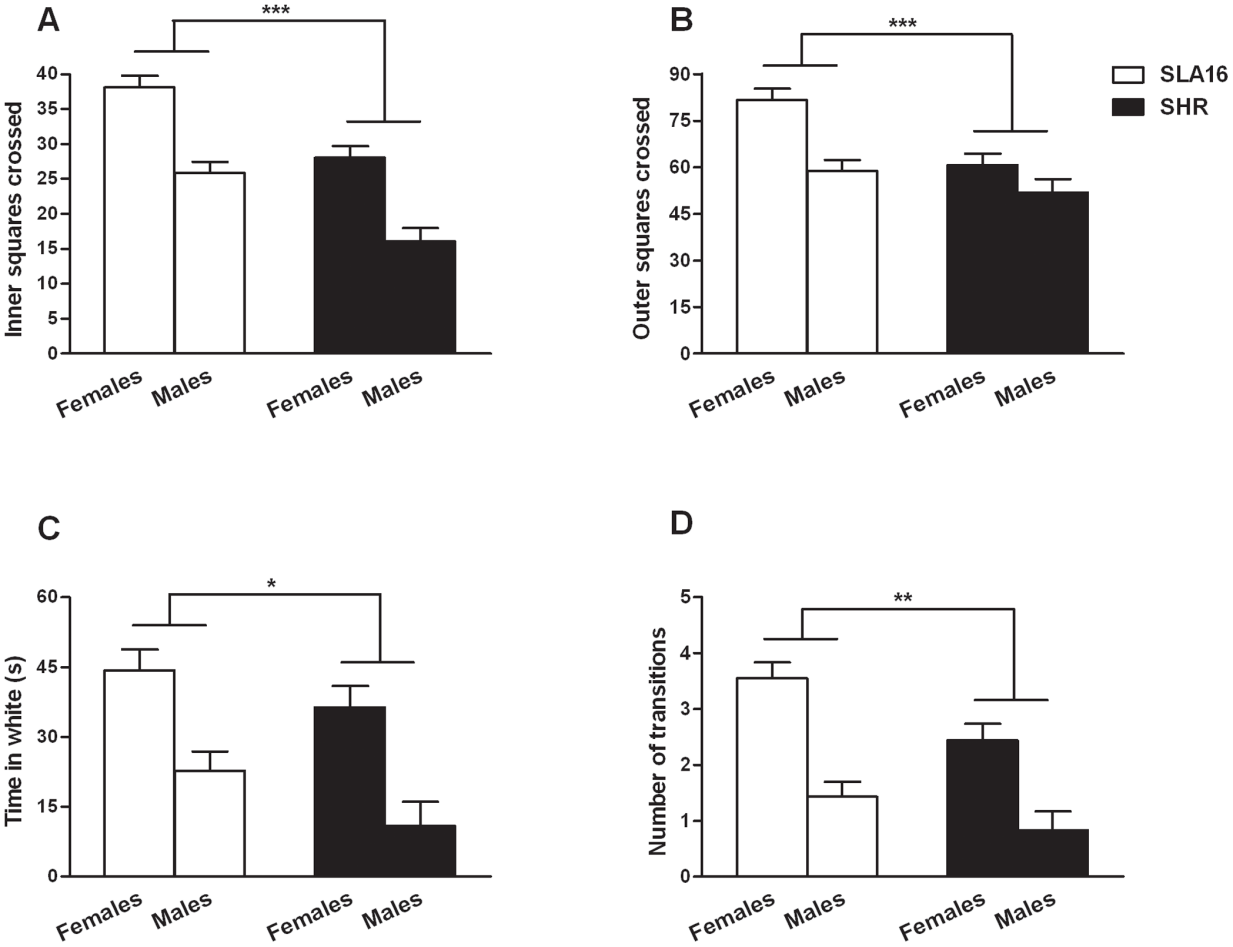
Open field and light/dark box test. Inner (A) and outer (B) locomotion in the open field test, time spent in the white compartment (C) and number of transitions between compartments (D) in the light/dark box test displayed by SLA16 and SHR rats from both sexes. Bars and vertical lines represent the means and S.E.M. of animals grouped by sex and strain (n = 18–28/sex/strain). (*), (**) and (***) represent significant (p<0.05, p<0.01 and p<0.001, respectively) overall effect of strain (two-way ANOVA).

The ANOVA also detected anxiety-related differences in the light/dark box (Figure 4C, D), with SLA16 rats (less anxious-like) spending more time in the light compartment (F_(1,89)_ = 4.58, p<0.05) and performing more transitions between compartments (F_(1,89)_ = 8.17, p<0.01) than SHRs. Again, overall sex differences were found for both measures, with females displaying higher scores. No strain-sex interactions were found in the open field and light/dark box.

**Figure 5:**
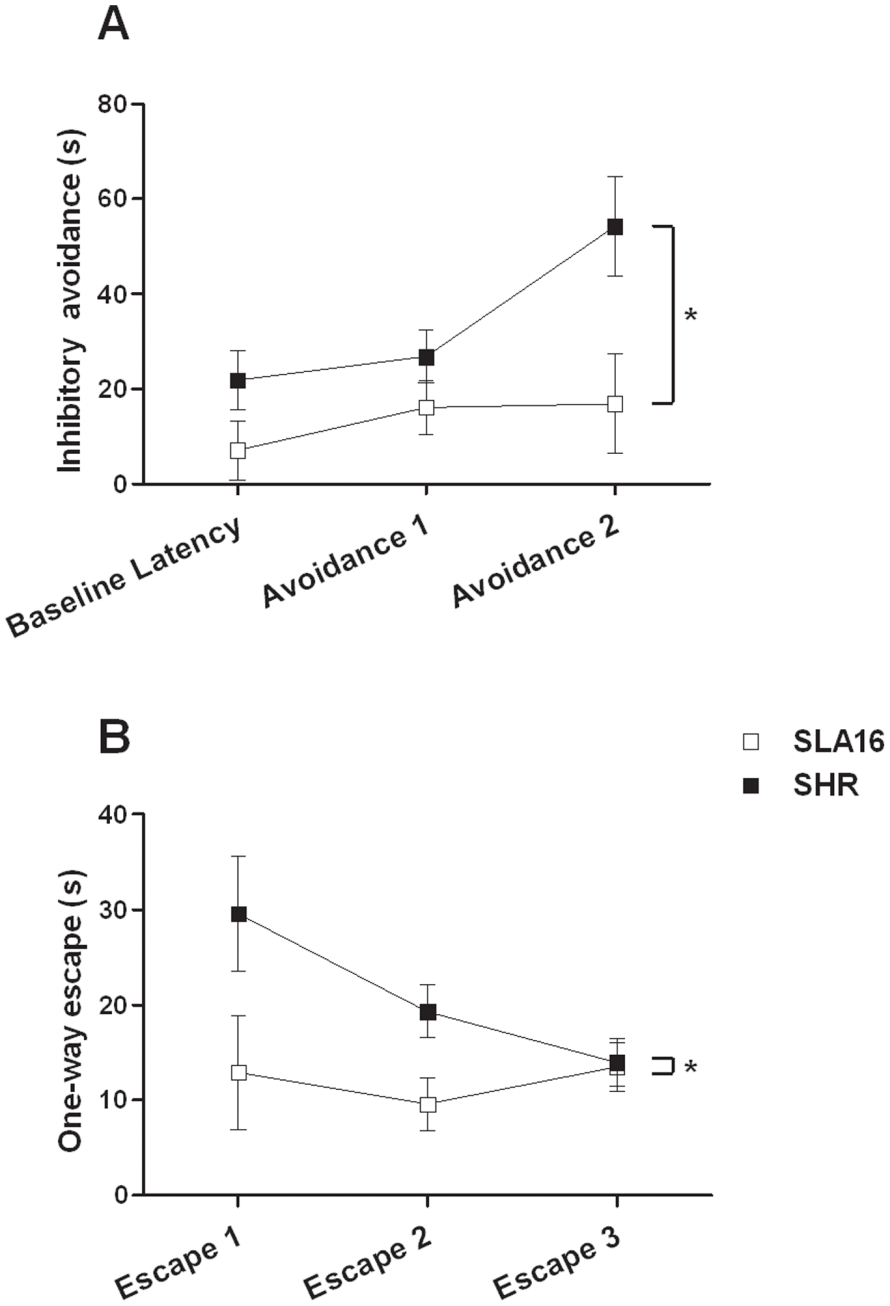
T-maze. Inhibitory avoidance and one way escape times (s) displayed by SLA16 and SHR male rats in the T-maze. Squares and vertical lines represent the means and S.E.M. of animals grouped by strain and trial (n = 12/strain). (*) represent significant (p<0.05) overall effect of strain (repeated-measure ANOVA).

The T-maze (Figure 5A, B) revealed differences both in inhibitory avoidance (model of generalized anxiety disorder) and one-way escape (model of panic disorder). For inhibitory avoidance, the repeated-measure ANOVA revealed a main effect of genotype (F_(1,22)_ = 7.12, p<0.05), with SLA16 exhibiting lower latencies (thus a less anxious profile) than SHR. For one-way escape, the ANOVA showed that SLA16 rats escaped from the open arms more quickly than SHRs (F_(1,22)_ = 4.53, p<0.05), suggesting that, in spite of the congenic strain being less anxious than SHRs, it also shows more “panic-like” behavior.

### Locomotor Genetic Effects are Novelty-dependent

**Figure 6:**
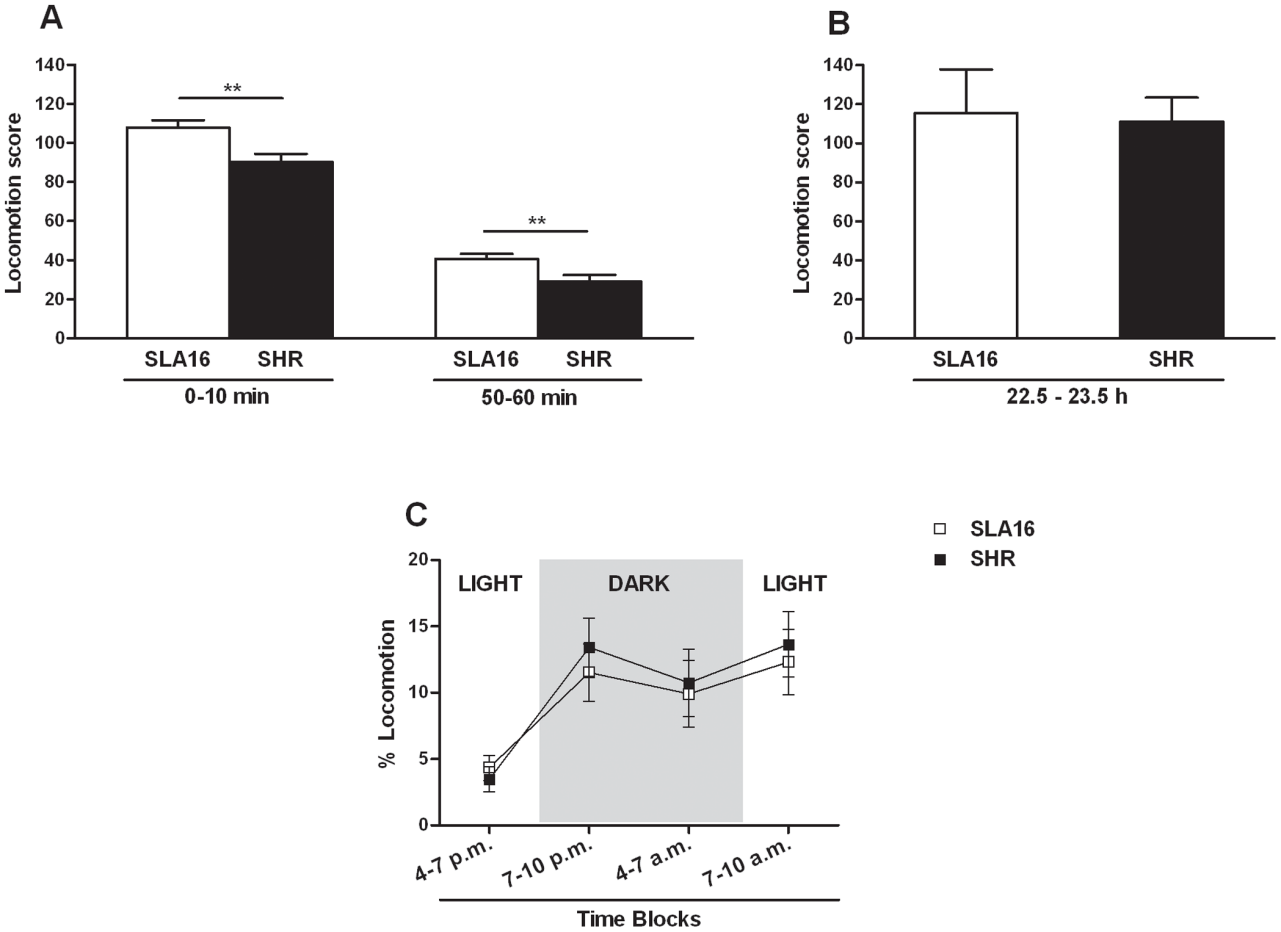
Locomotion scores in the activity and home-cages. Locomotor activity scores displayed by SLA16 and SHR rats in the unfamiliar (A, both sexes pooled together) and familiar (B, males only) activity cages, and in the home-cage behavior test (C, both sexes pooled together ). In A and B bars and vertical lines represent the means and S.E.M. of animals grouped by strain (n = 38–50/strain for the unfamiliar activity cage, n = 10/strain for the familiar activity cage), while in C squares and vertical lines represent the means and S.E.M. of animals grouped by strain and time block (n = 11 cages/group). (**) represent significant (p<0.01) overall effects of strain (two-way ANOVA for the activity cages and repeated measures ANOVA for the home-cage behavior test).

As shown in Figure 6A, locomotor differences between genotypes were found in the unfamiliar activity cage both in the first (0–10) and last (50–60) 10-min periods (F_(1,84)_ = 9.98, p<0.01; F_(1,84)_ = 7.32 p<0.01, respectively). Nevertheless, after a period of 22.5 h of habituation in the familiar cage (Figure 6B), this difference disappeared (p>0.05), thus suggesting that the higher activity of congenic rats found in the open field and unfamiliar activity cage reflects a lower emotional reaction to novelty, rather than a spontaneous hyperactivity. Reinforcing these data, the home-cage behavior test showed no differences between strains in the frequency of any of the behaviors observed (F_(3, 60)_ = 0.21, p>0.05) during 6 h of darkness and 6 h of light, including locomotion (Figure 6C), regardless of sex or the time blocks analyzed.

### No Attentional Deficits were Detected in Congenic Rats

**Figure 7:**
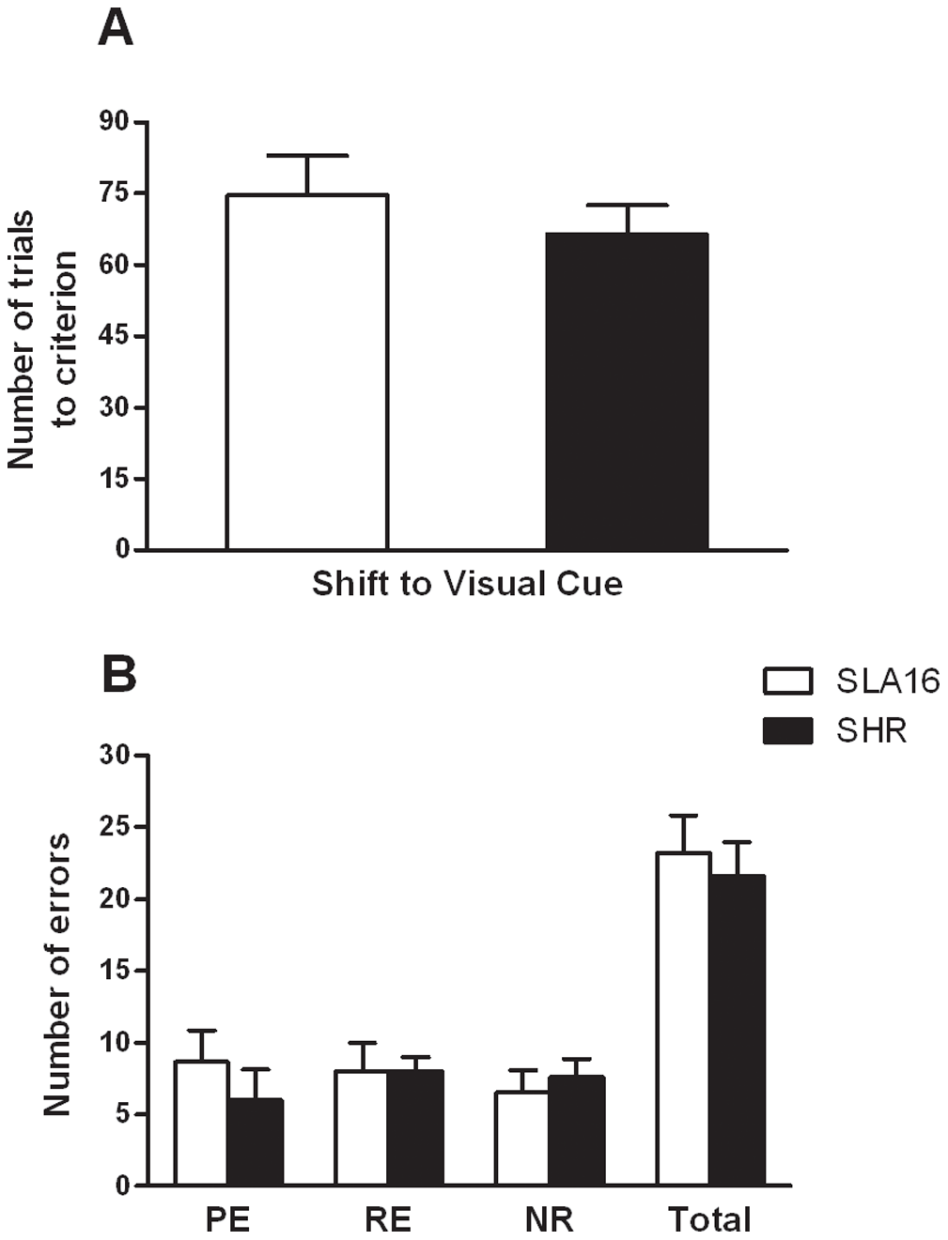
Set-shifting procedure. Number of trials to criterion (A) and number and types of errors (B) of SLA16 and SHR male rats during the visual cue discrimination task, in the set-shifting procedure. Bars and vertical lines represent the means and S.E.M. of rats grouped by strain (n = 9–12/strain). No significant (p>0.05) effect of strain was detected for the number of trials to criterion and number of perseverative (PE), regressive (RE), never-reinforced (NR) or total errors (Student’s t-test).

A t-test revealed that SLA16 and SHR strains did not differ in the attentional set-shifting procedure (Figure 7A, B), either in the number of trials to reach the learning criterion or in the number and type of errors committed during the visual cue discrimination task (p>0.05), indicating that these strains behave similarly in attention-related aspects.

### Behavioral Differences between Genotypes are Confirmed in the Triple Test

**Figure 8:**
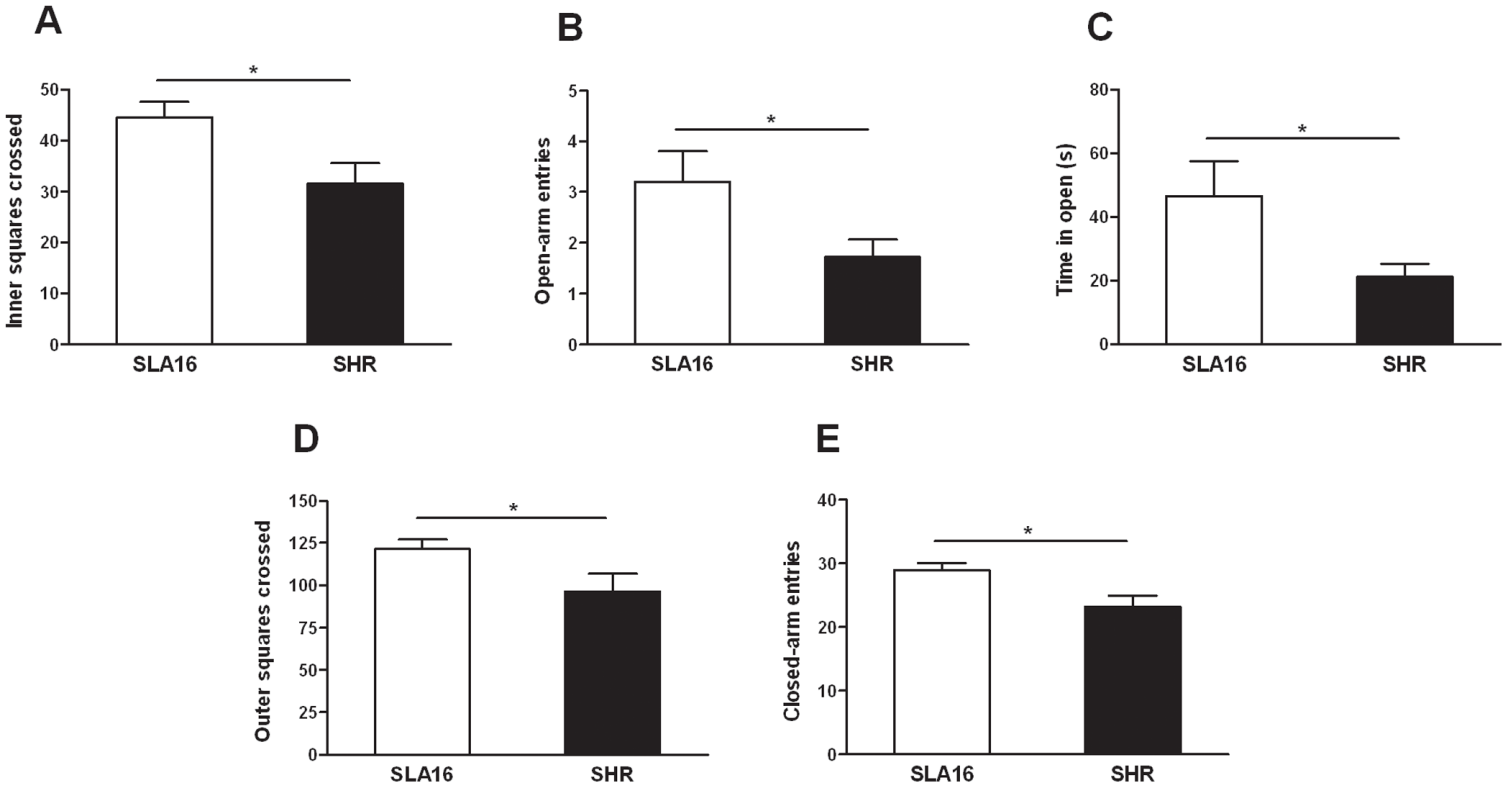
Exploration levels displayed in the triple test. Anxiety- (A, inner locomotion in the open field test, B, number of entries and C, time spent in the open arms in the elevated plus maze test) and locomotion-related (D, outer locomotion in the open field test and E, number of entries in the closed arms in the elevated plus maze test) behaviors displayed by SLA16 and SHR male rats in the triple test. Bars and vertical lines represent the means and S.E.M. of animals grouped by strain (n = 14–15/strain; one outlier from the SHR strain was not included in the analysis). (*) represent significant (p<0.05) effect of strain (Student’s t-test).

In the triple test, anxiety- (Figure 8A, B, C) and locomotion-related (Figure 8D, E) differences between strains were confirmed in the open field and elevated plus maze sections. T-tests showed that open field inner (p<0.05) and outer (p<0.05) locomotion, and number of entries (p<0.05) and time (p<0.05) spent in the open arms (p<0.05) as well as the number of closed-arm entries in the elevated plus maze were higher for SLA16 rats, in comparison to SHRs, confirming that the former seems less anxious and more active in novel environments than the latter.

### Anxiety-related Differences do not Depend on the Estrous Cycle

**Figure 9:**
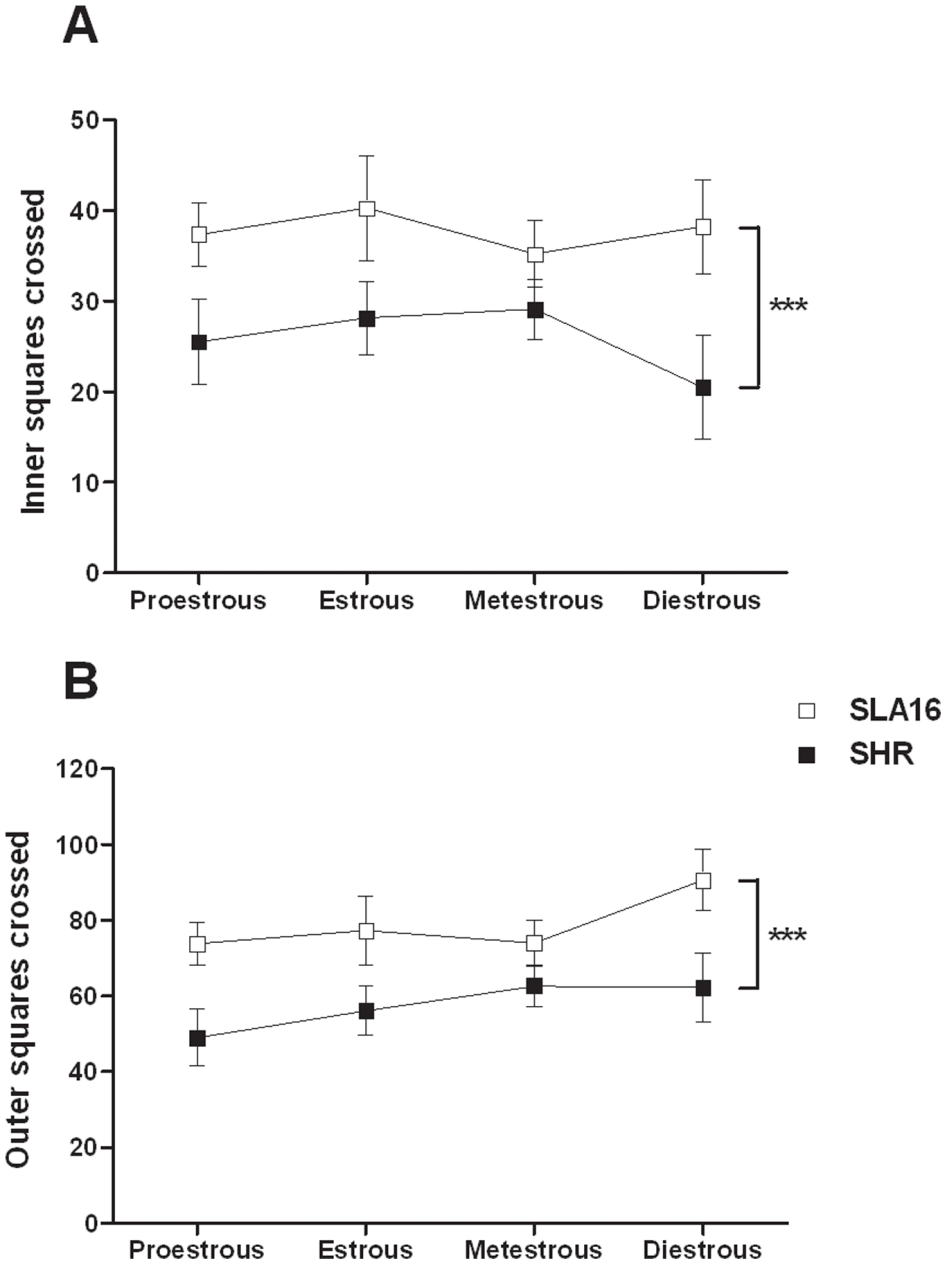
Effects of the estrous cycle phases on behaviors in the open field. Inner (A) and outer (B) locomotion displayed by SLA16 and SHR female rats. Squares and vertical lines represent the means and S.E.M. of animals grouped by estrous cycle phase and strain (n = 4–12/phase/strain). (***) represent significant (p<0.001) overall effect of strain (two-way ANOVA).

Because a previous study [16] suggested that the effects of *Anxrr16* in females depended on their estrous cycle, open field tests were also run in cycle-controlled females. A two-way ANOVA (Figure 9A, B) revealed again an overall effect of the genotype for inner F_(1,52)_ = 13.53, (p<0.001) and outer F_(1,52)_ = 17.34, (p<0.001) locomotion, but no effect of the phase or interaction between phase and genotype were observed.

## Discussion

A primary goal of this study was to isolate and confirm the effects of a rat genome region named *Anxrr16* on experimental anxiety. The development of the congenic SLA16 strain allowed us to confirm the location of *Anxrr16* on chromosome 4, within a region of 86.3 Mpb between the molecular markers D4Rat76 and D4Mgh11. Our results also confirm the transgressive and counterintuitive nature of its effects. “Transgressive segregation” is a term that refers to the recombination of parental chromosomes in the offspring which generates phenotypes outside the normal range of variation observed in either parental gene pool [9]. This is a relatively common phenomenon in QTL studies [26], [27], [28] and can be due to numerous factors, including epistasis, the additive effects of multiple recombined *loci* and the unmasking of recessive alleles that were masked in the parents [9], [29].

The present results thus confirmed our hypothesis that the anxiety levels of the new congenic animals would be reduced to an even lower level than those found in the parental SHR strain. Such behavioral effects were herein demonstrated in SLA16 rats, which showed higher scores of central locomotion in the open field test when compared to SHR controls. This effect was observable in both sexes, did not depend on the estrous cycle and was present under different lighting conditions. The anxiolytic-like effects of LEW alleles in this chromosome segment were corroborated in other anxiety tests, such as the light/dark box, T-maze, and triple test. In all these paradigms, which vary in the nature and intensity of anxiogenic stimuli, SLA16 rats behaved even calmer than SHR rats, known for their low anxiety profile.

Because this portion of chromosome 4 also affected indices of locomotion in different unfamiliar environments (open field, triple test and activity cage), we cannot discard the possibility that this genome region may have influenced, to some extent, something other than anxiety (e.g. motor activity). Yet, in familiar activity cages as well as in their home-cages, no strain differences appeared.

Although outer locomotion in the open field is often considered a measure of motor activity, this behavior is also modulated by anxiety levels [30], [14], [31]. In the open field, as in many other exploration-based anxiety tests, it is not possible to completely sort apart motor- and emotionality-related behaviors [32]. Therefore, the present effect of *Anxrr16* on open field peripheral locomotion, although not confirming the results by Ramos et al. [12], is consistent with previous observations showing a genetic correlation between central and peripheral locomotion in the open field [33], [14], [16]. Since no strain differences were seen either in the activity scores following 22.5 h of familiarization or in their spontaneous home-cage behavior, and because anxiety-related effects were also found in behaviors that do not involve a locomotor component (i.e. time spent in the white compartment of the light/dark box and in the open arms of the EPM and triple test), the present results suggest that *Anxrr16* modulates emotional reactivity, rather than spontaneous motor activity.

Concerning attention-related measures, SLA16 did not differ from SHR in behavioral flexibility or in the ability to establish and maintain a new learning strategy, as indicated by the number of trials to reach the criterion or the number and type of errors committed during the visual cue discrimination task. These findings do not indicate that the increased activity displayed by SLA16 in novel environments is related to ADHD, even though SHR rats are widely used as a model of this pathology.

Different aspects of the behavioral effects of *Anxrr16* could be observed in the triple test, an integrated test that was conceived to provide a wide range of anxiety- and locomotion-related measures in one single trial [23], [34], [35]. In the open field and elevated plus maze parts of the triple test, differences between SLA16 rats and their controls appeared in the expected direction, thus showing their hypo-anxious and hyperactive profile when exposed to novel environments. Curiously, no differences were detected in the light/dark box, which had revealed strain differences in its individual form, maybe due to the much larger sample size used in the latter protocol.

Originally, *Anxrr16* had been detected only in females [12], whereas Izídio et al. [16] found that it could affect males and, in the case of females, its effects seemed to be modulated by the estrous cycle. The present results showed that *Anxrr16* has a robust effect in both males and females over all phases of the estrous cycle. Since the congenic strain SLA16 is a much “cleaner” tool when compared to the F2 populations used in the previous studies, because it reveals the expression of the target *locus* without the interference of the rest of the genome, it may have allowed the detection of genetic differences that could not be consistently detected before.

To our knowledge, this is the first report of an anxiety-related *locus* being isolated in the genome of rats through the construction of a congenic strain. Considering the exceptional power of this particular QTL (second strongest ever described), this new tool will be extremely useful to dissect and further reduce the size of this chromosome segment, possibly through the development of congenic sub-lines [36]. The identification of genes through the use of congenic sub-lines has been successfully reported, including genes such as *Mpdz* (influencing the predisposition to alcohol physiological dependence) [37], and *Repin1* (associated to metabolic syndromes) [38]. Furthermore, studies involving comparative genomics, gene expression and validation of candidate genes shall lead to the discovery of the molecular pathways underlying the present anxiety-related behaviors. Differently from knockout and transgenic approaches, the present strategy is based on natural variations in genes and gene products that physiologically modulate behavior, in a manner that may ultimately facilitate translation from animal models to human clinical conditions.

In summary, the present study confirms the trangressive and counterintuitive effect of *Anxrr16* on anxiety-related behaviors, which was made possible through the construction of a congenic rat strain for this *locus*, named SLA16. This QTL also influenced novelty-dependent locomotion, but not spontaneous locomotion or indices of attention. The anxiety levels of the non-anxious SHR strain were further decreased by the introgression of LEW alleles in the *Anxrr16* region. This clearly demonstrates how phenotypes that are apparently extreme for a given polygenic trait may hide major opposite-acting genes, which may remain forever concealed if scientists reproduce the over-simplified strategy of “looking for bad genes only in bad phenotypes”.
